# BRD4 inhibition suppresses cell growth, migration and invasion of salivary adenoid cystic carcinoma

**DOI:** 10.1186/s40659-017-0124-9

**Published:** 2017-05-25

**Authors:** Limei Wang, Xiuyin Wu, Ruolin Wang, Chengzhe Yang, Zhi Li, Cunwei Wang, Fenghe Zhang, Pishan Yang

**Affiliations:** 1 Department of Periodontology, School of Stomatology, Shandong University, 44-1 West Wen Hua Road, Jinan, 250012 Shandong People’s Republic of China; 2 Shandong Provincial Key Laboratory of Oral Tissue Regeneration, Jinan, 250012 Shandong China; 3 Department of Stomatology, Laiwu City People’s Hospital, Laiwu, 271100 Shandong China; 4 Department of Oral & Maxillofacial Surgery, Qilu Hospital, and Institute of Stomatology, Shandong University, Jinan, 250012 Shandong China; 5 Department of Prosthodontics, School of Stomatology, Shandong University, Jinan, 250012 Shandong China; 6 Department of Oral & Maxillofacial Surgery, School of Stomatology, Shandong University, 44-1 West Wen Hua Road, Jinan, 250012 Shandong People’s Republic of China

**Keywords:** Salivary adenoid cystic carcinoma, BRD4 inhibition, Proliferation, Migration, Epithelial–mesenchymal transition

## Abstract

**Background:**

Bromodomain-containing protein 4 (BRD4) inhibition is a new therapeutic strategy for many malignancies. In this study, we aimed to explore the effect of BRD4 inhibition by JQ1 on in vitro cell growth, migration and invasion of salivary adenoid cystic carcinoma (SACC).

**Methods:**

The human normal epithelial cells and SACC cells (ACC-LM and ACC-83) were treated with JQ1 at concentrations of 0, 0.1, 0.5 or 1 μM. Cell Counting Kit-8 (CCK-8) assay was performed to evaluate cell proliferation. Cell apoptosis and cell cycle distribution was evaluated by Flow cytometry. Immunofluorescence staining was used to examine the expression of BRD4 in SACC cells. The quantitative real-time polymerase chain reaction (qRT-PCR) assay and western blot assay were performed to examine messenger RNA (mRNA) and protein levels in SACC cells. Wound-healing assay and transwell assay were used to evaluate the activities of migration and invasion of SACC cells.

**Results:**

JQ1 exhibits no adverse effects on proliferation, cell cycle and cell apoptosis of the normal human epithelial cells, while suppressed proliferation and cell cycle, and induced apoptosis of SACC cells, down-regulated the mRNA and protein levels of BRD4 in SACC cells, meanwhile reduced protein expressions of c-myc and BCL-2, two known target genes of BRD4. Moreover, JQ1 inhibited SACC cell migration and invasion by regulating key epithelial–mesenchymal transition (EMT) characteristics including E-cadherin, Vimentin and Twist.

**Conclusions:**

BRD4 is an important transcription factor in SACC and BRD4 inhibition by JQ1 may be a new strategy for SACC treatment.

## Background

Salivary adenoid cystic carcinoma (SACC) is a highly malignant carcinoma that most often arises from the secretory epithelial cells of salivary glands [1] and comprises approximately 10% of all salivary tumors [2]. SACC is characterized by several unique properties, such as slow growth; however, high rates of local recurrence, tendency to perineural invasion and distant metastases [1, 3, 4]. Extensive effort has been performed to develop many strategies for management of SACC patients, but the 15-year survival is only 25% [5]. Therefore, it is urgent to develop an efficient therapy against this carcinoma to improve patient outcome.

Recent studies have demonstrated that epigenetic regulators are becoming new therapeutic targets for cancer therapy [6], of which as a member of the bromodomain and extraterminal domain (BET) proteins, bromodomain-containing protein 4 (BRD4) has been widely investigated. It has been reported that BRD4 is associated with a variety of cancers due to its role in the regulation of cell cycle progression [7–12]. Studies of yeast and mammalian BET family proteins, including BRD4, indicate that they recognize acetylated chromatin in vivo and regulate the expression of important oncogenes, for example, c-myc and BCL-2 [13]. Besides, BRD4 can also interact with the positive transcription elongation factor b (p-TEFb) to lead to phosphorylation of its unique C-terminal domain (CTD), thus allowing the productive elongation of c-myc and BCL-2 [14]. Further study indicates that BRD4 has a vital role in promoting cell cycle progression from G0 to G1 and entry into S phase. BRD4 knockdown cells are growth impaired and grow more slowly than control cells accompanied by decrease in G1 gene expression [15]. Moreover, the recruitment of BRD4 was requisite in Twist-mediated epithelial–mesenchymal transition (EMT) [16]. Consequently, BRD4 plays important roles in genesis, development and metastasis of tumors.

BRD4 has been validated as a therapeutic target in many malignant tumors, including hepatocellular carcinoma (HCC), leukaemia, osteosarcoma, pancreatic cancer and so on [7, 9, 17–26]. The small molecule compound JQ1 first reported by Filippakopoulos and colleagues is of particular interest among the inhibitors of BRD4 and can competitively displace BRD4 from acetylated histones [27]. It has been reported that JQ1 treatment inhibited proliferation of Ewing sarcoma cells in vitro and reduced tumor growth in vivo in a dose dependent manner [25]. Inhibition of BRD4 in thyroid cancer cells by JQ1 has been demonstrated to decrease cell viability in vitro and suppress tumor growth in vivo [8]. Similar inhibitory effect of JQ1 was observed in many other malignant tumors including melanoma, HCC and ovarian cancer [9, 10, 26]. However, the effect of JQ1 on the growth and invasion of SACC has not been well investigated.

In this study, we investigated the effect of BRD4 inhibition by JQ1 on cell growth, migration and invasion of SACC cells in vitro, so as to develop a new therapeutic target for SACC.

## Results

### JQ1 exhibits no adverse effects on proliferation, cell apoptosis and cell cycle of the human normal epithelial cells

**Fig. 1:**
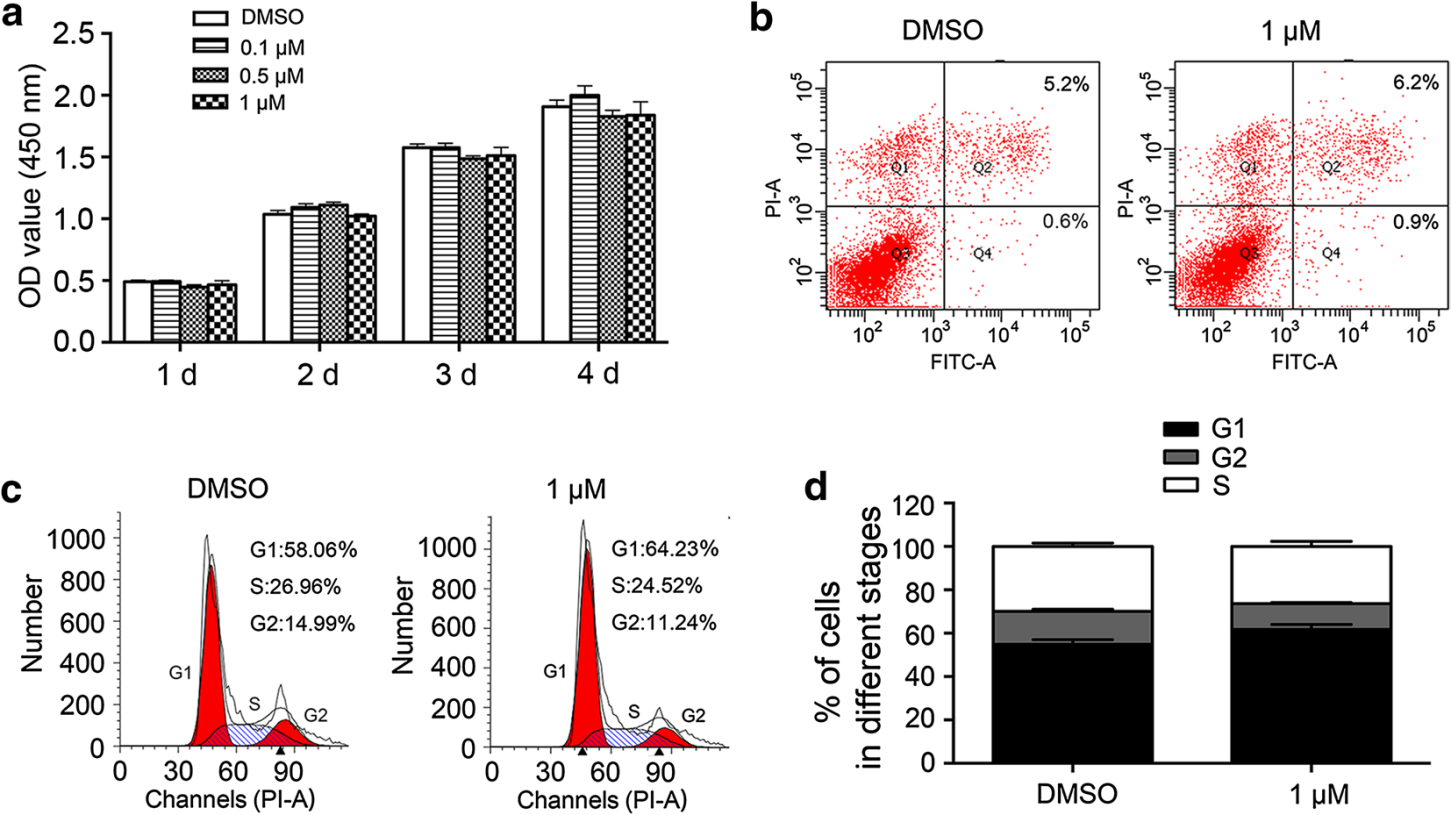
JQ1 exhibits no adverse effects on proliferation, apoptosis and cell cycle of the human normal epithelial cells. **a** The proliferation of ACC-LM and ACC-83 cells after JQ1 treatment for 1–4 days; **b** apoptosis of the human normal epithelial cells treated with JQ1 at concentration of 1 µM for 48 h; **c** the cell cycle of the human normal epithelial cells after JQ1 treatment at the concentration of 1 µM for 48 h; **d** the fractions of the human normal epithelial cells in each phase of the cell cycle after JQ1 treatment at the concentration of 1 µM for 48 h

Firstly, we investigated the effects of JQ1 at various concentrations on proliferation, cell apoptosis and cell cycle of the human normal epithelial cells. No significant changes were found in cells treated with JQ1 when compared with the control cells (Fig. 1). These data revealed that JQ1 has no adverse effects on the growth of the human normal epithelial cells.

### JQ1 reduces SACC cell proliferation

**Fig. 2:**
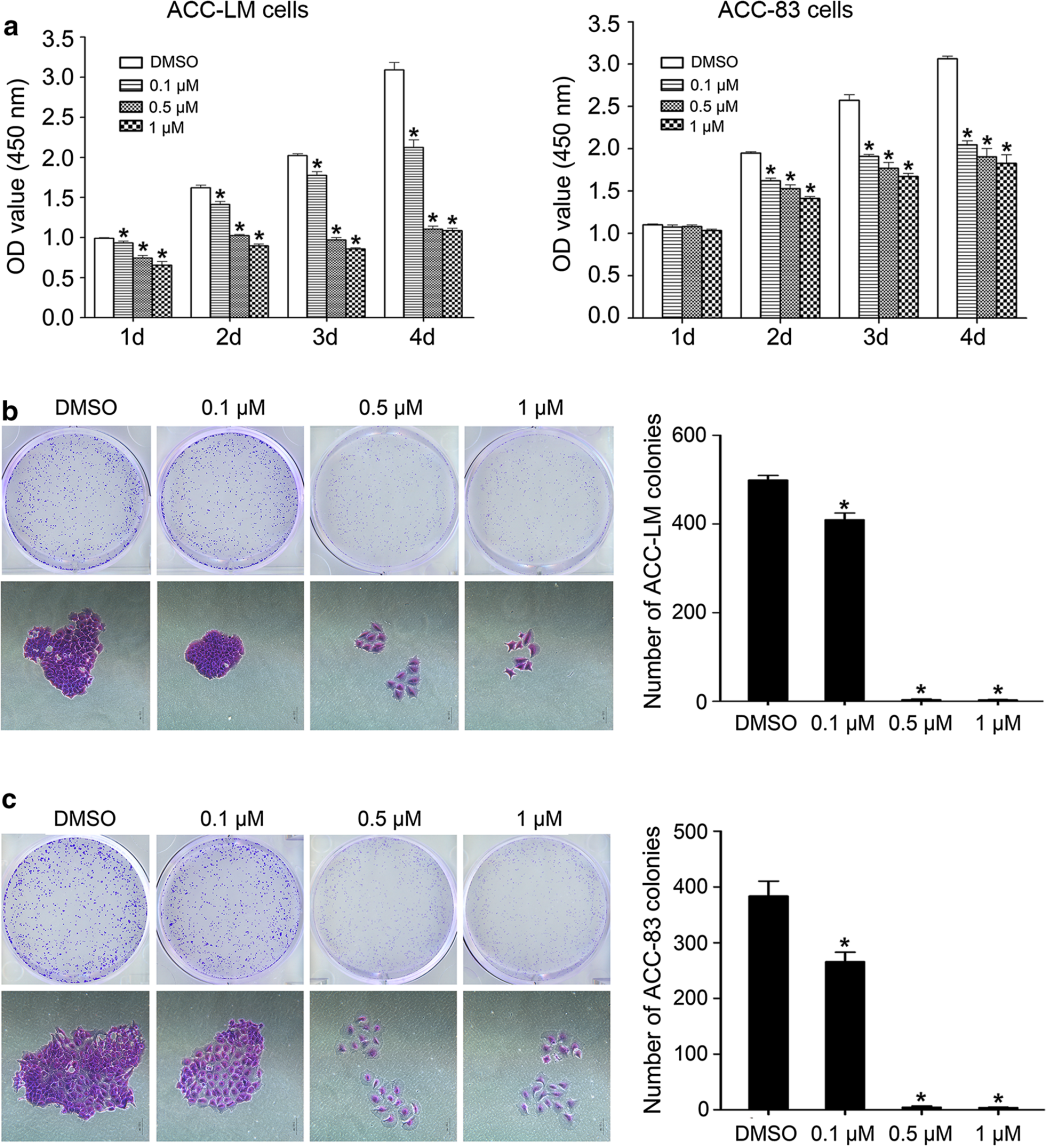
JQ1 reduces the growth of SACC cells. **a** The proliferation of ACC-LM and ACC-83 cells after JQ1 treatment for 1–4 days; Macroscopic and microscopic (×100) images of colonies formed by ACC-LM (**b**) and ACC-83 (**c**) cells treated with JQ1 for 7 days. *P < 0.05 vs. the control group (the DMSO group)

CCK-8 assay was performed to evaluate the effect of JQ1 on the proliferation of SACC cells. The results showed that JQ1 significantly inhibited proliferation of ACC-LM cells when compared with the control group throughout the duration of the experiment (Fig. 2a). The proliferation of ACC-83 cells at day 1 had no significant change when compared with the control group. However, the proliferation of ACC-83 cells was significantly decreased after JQ1 treatment at day 2–4 (Fig. 2a).

To confirm above results, colony formation assay was performed to further clarify the antiproliferative effects of JQ1 on SACC cells. As expected, the number and size of colonies of ACC-LM and ACC-83 cells decreased sharply in the presence of JQ1 at various concentrations (Fig. 2b, c). In fact, the groups treated by 0.5 and 1 µM of JQ1 had no colony formation. Therefore, the results suggest that inhibition of BRD4 by JQ1 has potent antiproliferation effects on SACC.

### JQ1 induces apoptosis and suppresses cell cycle in SACC cells

**Fig. 3:**
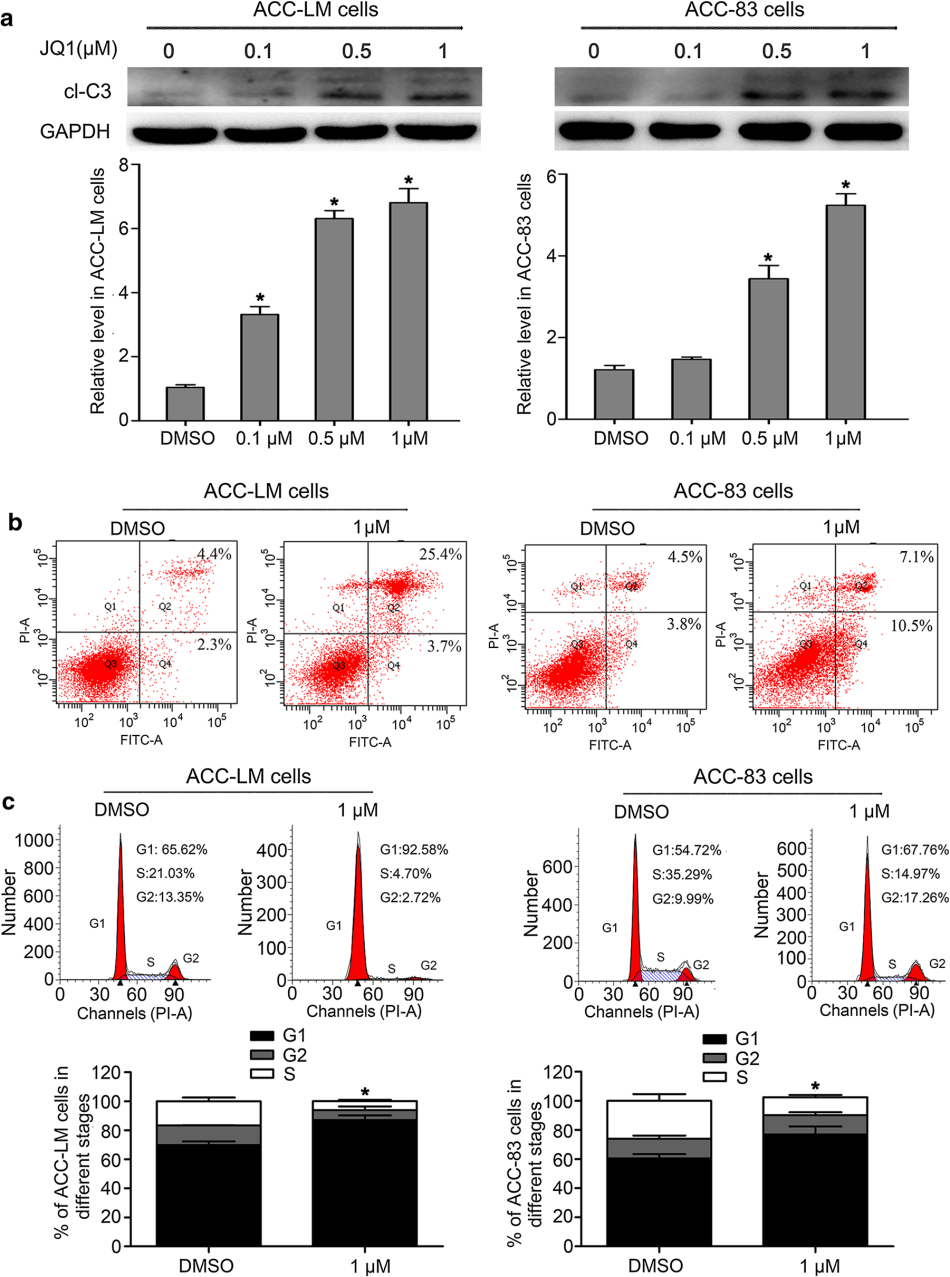
JQ1 induces apoptosis and inhibits cell cycle in SACC cells. **a** The protein levels of cleaved cl-C3 in ACC-LM and ACC-83 cells treated with JQ1 at various concentrations for 48 h; **b** apoptosis of ACC-LM cells and ACC-83 cells treated with JQ1 at concentration of 1 µM for 48 h; **c** the fractions of ACC-LM cells and ACC-83 cells in each phase of the cell cycle are shown after JQ1 treatment at the concentration of 1 µM for 48 h. *P < 0.05 vs. the control group (the DMSO group). *cl-C3* cleaved caspase-3

To identify the mechanism of the antiproliferation effects of JQ1 in SACC cells, the cell cycle and apoptosis status of SACC cells in the presence of JQ1 were analyzed. The results showed that the protein levels of cleaved caspase-3 (cl-C3) were significantly increased in ACC-LM cells treated with JQ1 at various concentrations within 48 h in comparison with the control group (Fig. 3a). JQ1 at concentrations above 0.1 µM significantly up-regulated the levels of cl-C3 in ACC-83 cells (Fig. 3a). The results of flow cytometry showed that JQ1 significantly increased the percentage of apoptotic ACC-LM and ACC-83 cells (Fig. 3b), which was consistent with the protein levels of cl-C3. To further investigate the antiproliferation effects of JQ1 on SACC cells, the cell cycle was detected. We found that JQ1 treatment at various concentrations for 48 h led to a decreased percentage of ACC-LM and ACC-83 cells in the S phase (Fig. 3c). Taken together, these results reveal that JQ1 may inhibit the proliferation of SACC cells via inducing apoptosis and suppressing cell cycle.

### JQ1 inhibits BRD4 expression

**Fig. 4:**
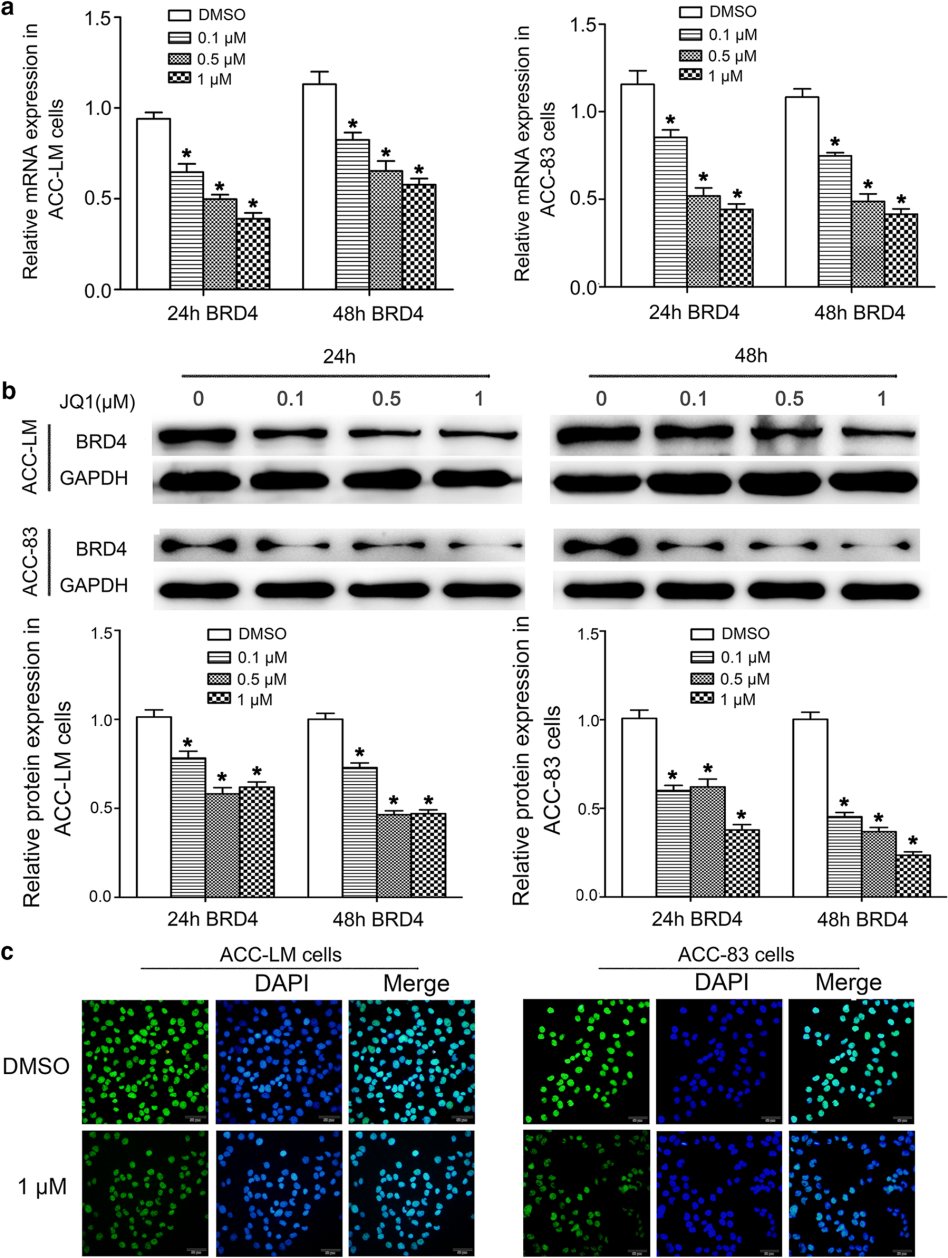
JQ1 inhibits BRD4 expression in SACC cells. **a** The mRNA levels of BRD4 in ACC-LM and ACC-83 cells treated with JQ1 for 24 and 48 h. **b** The protein levels of BRD4 in ACC-LM and ACC-83 cells treated with JQ1 for 24 and 48 h; **c** immunofluorescence staining of BRD4 in ACC-LM and ACC-83 cells treated with JQ1 at the concentration of 1 µM for 24 h (×200). *P < 0.05 vs. the control group (the DMSO group)

We investigated the effect of JQ1 on BRD4 expression in ACC-LM and ACC-83 cells. The results of qRT-PCR and western blot assays showed that the expression levels of BRD4 were significantly decreased in cells treated with JQ1 (Fig. 4a, b). In addition, the results of immunofluorescence staining also showed that the expression of BRD4 was inhibited in ACC-LM and ACC-83 cells after treated with JQ1 for 24 h (Fig. 4c).

### JQ1 inhibits protein expression of Cyclin D1, c-myc and BCL-2

**Fig. 5:**
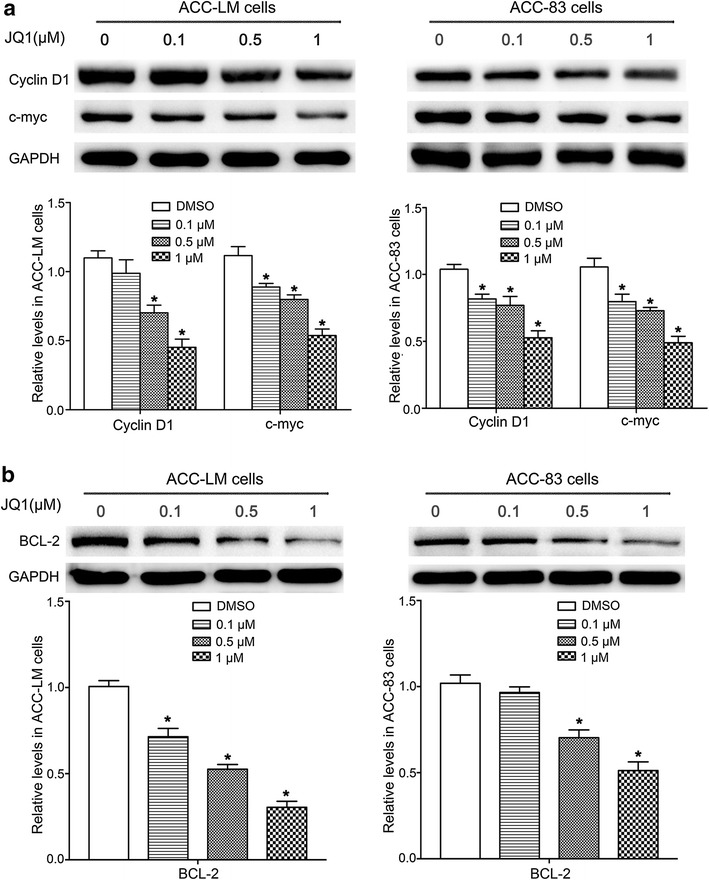
JQ1 down-regulates Cyclin D1, c-myc and BCL-2 expressions in SACC cells. **a** The protein levels of Cyclin D1 and c-myc in ACC-LM cells and ACC-83 cells treated with JQ1 at various concentrations for 48 h; **b** the protein levels of BCL-2 in ACC-LM cells and ACC-83 cells treated with JQ1 at various concentrations for 48 h. *P < 0.05 vs. the control group (the DMSO group)

Cyclin D1 protein is associated with cell cycle and tumor progression. Considering the effect of JQ1 on cell cycle, we then evaluated the protein expression of Cyclin D1 in SACC cells after JQ1 treatment. We found that the protein levels of Cyclin D1 were significantly decreased in ACC-LM cells treated with JQ1 at concentration of 0.5 and 1 µM (Fig. 5a). Similarly, the protein levels of Cyclin D1 were significantly inhibited in ACC-83 cells after 48 h treatment by JQ1 at various concentrations (Fig. 5a).

Protein levels of c-myc and BCL-2, two known targets for BRD4, were also analyzed in SACC cells treated with JQ1. The results showed that the expression of c-myc was significantly decreased in ACC-LM and ACC-83 cells treated with JQ1 at 0.1, 0.5 and 1 µM (Fig. 5a). The protein levels of BCL-2 were also significantly down-regulated in ACC-LM cells treated with JQ1 at various concentrations (Fig. 5b). In ACC-83 cells, the expression levels of BCL-2 were significantly inhibited after treated with JQ1 at 0.5 and 1 µM (Fig. 5b). These data indicated that JQ1 inhibited expressions of targets for BRD4 in SACC cells.

### JQ1 inhibits the migration and invasion of SACC cells

**Fig. 6:**
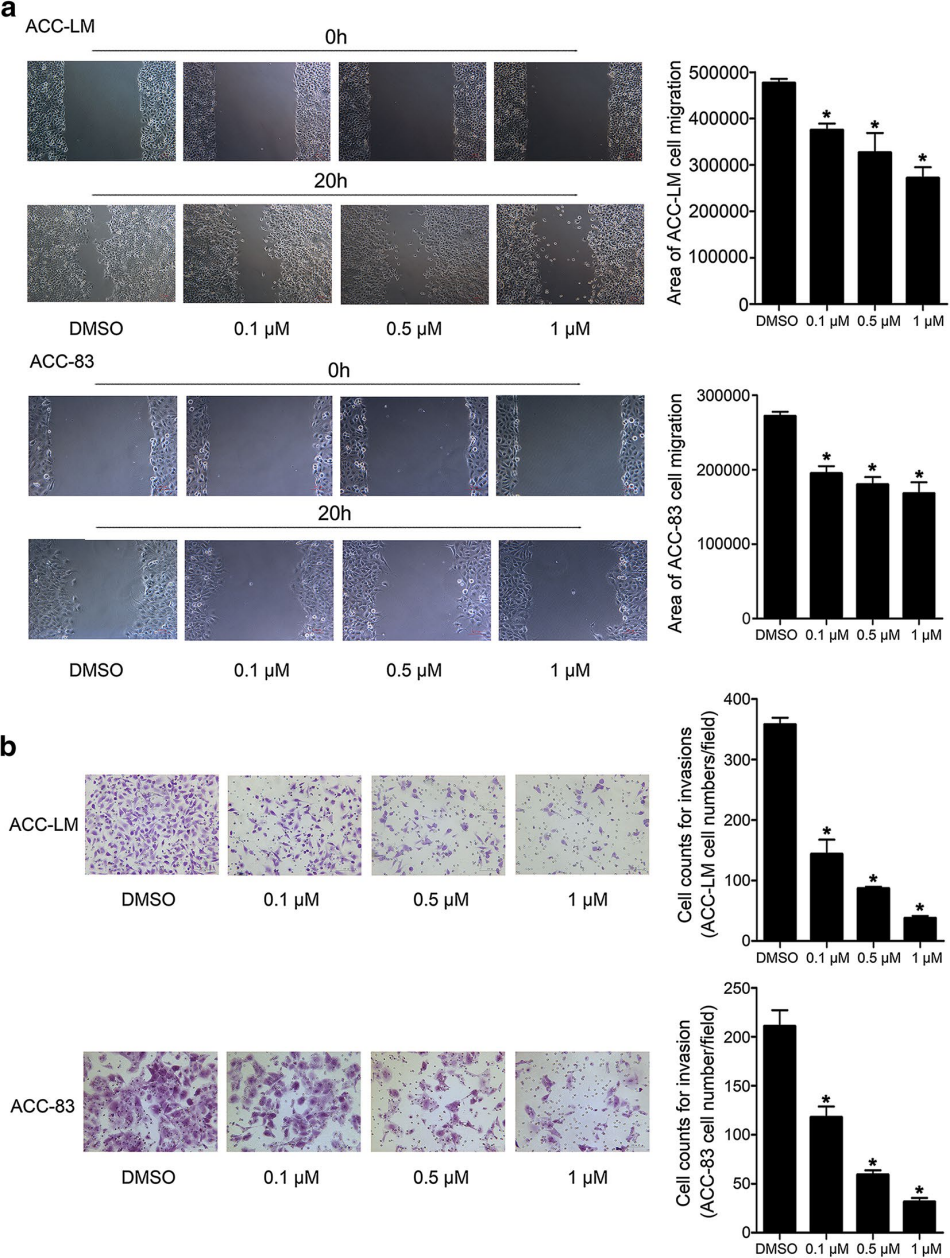
JQ1 inhibits the migration and invasion of SACC cells. **a** The migration of ACC-LM cells and ACC-83 cells treated by JQ1 at various concentrations for 20 h (×100); **b** the invasion of ACC-LM cells and ACC-83 cells treated by JQ1 at various concentrations for 20 h (×200). *P < 0.05 vs. the control group (the DMSO group)

The effect of JQ1 on cell migration and invasion was investigated. The results of wound-healing assay showed that the areas covered by migrated ACC-LM and ACC-83 cells were significantly reduced after 20 h treatment with JQ1 at various concentrations in comparison with the control group (Fig. 6a). Moreover, transwell invasion assay showed that JQ1 significantly decreased the amount of ACC-LM and ACC-83 cells invaded from the upper surface to the lower surface of the transwell insert (Fig. 6b). Therefore, these data indicate that JQ1 inhibits migration and invasion of SACC cells in vitro.

### JQ1 represses the progression of EMT in SACC cells by regulating key EMT characteristics

**Fig. 7:**
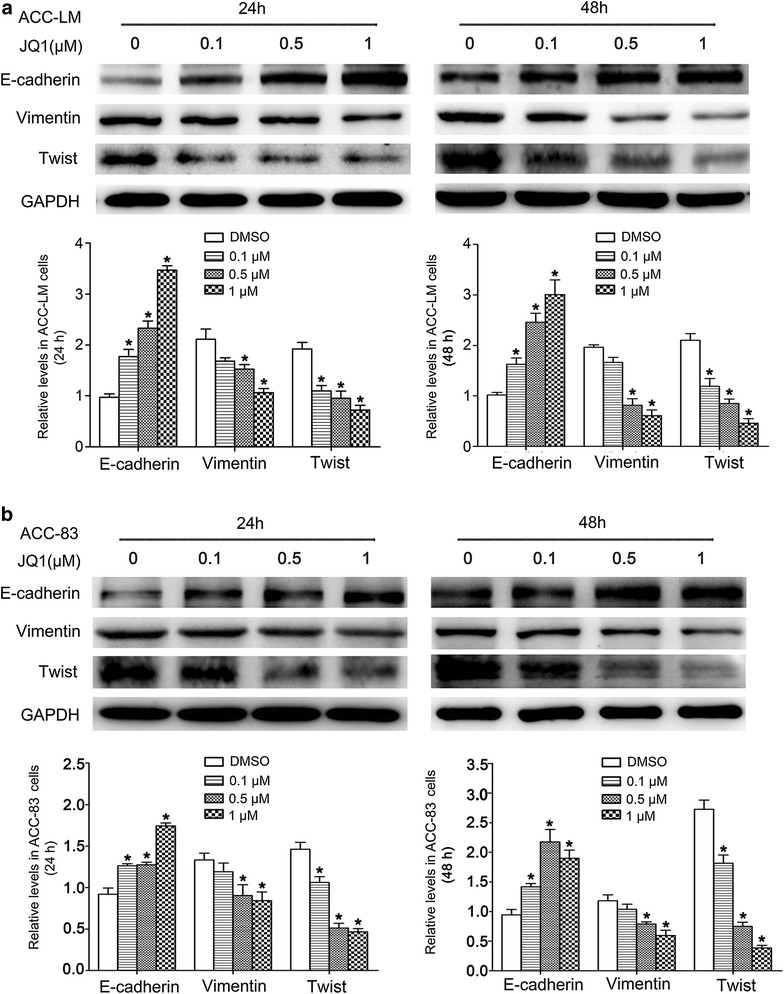
JQ1 represses several key EMT characteristics in SACC cells. The protein levels of E-cadherin, Vimentin and Twist in ACC-LM (**a**) and ACC-83 (**b**) cells treated with JQ1 for 24 and 48 h. *P < 0.05 vs. the control group (the DMSO group)

Epithelial–mesenchymal transition plays pivotal roles in tumor development and invasion, and is a key initiating event in the metastatic cascade. To investigate the molecular mechanism of the inhibition of JQ1 on migration and invasion of SACC cells, the levels of several EMT related proteins were examined. The results showed that the protein level of Twist was significantly repressed in ACC-LM and ACC-83 cells (Fig. 7) treated with JQ1 at various concentrations after 24 and 48 h when compared with the control group. Moreover, the protein levels of Vimentin were significantly down-regulated by JQ1 at concentrations of 0.5 and 1 µM in ACC-LM and ACC-83 cells (Fig. 7). The protein level of epithelial gene, E-cadherin, was up-regulated in ACC-LM and ACC-83 cells treated with JQ1 (Fig. 7). These results suggest that the inhibition effect of JQ1 on SACC cell migration and invasion may be due to EMT inhibition.

## Discussion

BRD4 inhibition suppresses growth and metastasis of several malignant tumors and BRD4 has been validated as a therapeutic target for tumor treatment [5, 26, 28, 29]. However, the effect of BRD4 inhibition on SACC has not been well investigated. In the present study, we found that the small molecular compound JQ1 had no adverse effects on the human normal epithelial cells, while inhibited SACC cell proliferation, migration and invasion, and down-regulated the expressions of BRD4, Cyclin D1, c-myc and BCL-2.

It has been demonstrated that BRD4 inhibition by JQ1 inhibits proliferation of many malignant tumors, including myeloma, melanoma, colorectal cancer, rhabdomyosarcoma and Ewing sarcoma [28, 30–32]. Therefore, we hypothesized that SACC cell proliferation might be inhibited by BRD4 inhibitor. As expected, our results showed that JQ1 repressed SACC cell proliferation and colony formation. As for antiproliferation mechanisms of JQ1, it has been reported that the antiproliferation activity of JQ1 in primary osteosarcoma cells was driven by the induction of apoptosis [33]. In addition, it has been demonstrated that BRD4 depletion in Hela cells induced G1 cell cycle arrest and apoptosis, and down-regulated the expression of Cyclin D1 [34]. As a patent inhibitor of BRD4, JQ1 may play the similar role as BRD4 depletion. In the present study, we found that JQ1 treatment decreased the percentage of S phase in SACC cells. The protein expression of cleaved caspase-3 and the percentage of apoptosis cells were significantly increased in SACC cells treated by JQ1. In addition, the expression of Cyclin D1 was significantly down-regulated in SACC cells treated with JQ1. Cyclin D1 has been found to be associated with tumor progression in numerous different tumor types [35]. All these data suggest that JQ1 may repress SACC cell proliferation via cell cycle arrest and inducing cell apoptosis.

Compared with the in situ overgrowth, distal metastasis is a more lethal property of malignant tumors [36]. SACC is characterized by strong invasion to peripheral nerves and high tendency to distant metastasis, which is a common cause of mortality in patients with this carcinoma. Therefore, approaches which can limit tumor invasion are urgent to be developed in SACC treatment. BRD4 gene plays an important role in tumor invasion. It has been demonstrated that high level of BRD4 promotes non-small cell lung cancer progression [37]. The suppression of BRD4 by a short hairpin RNA resulted in impaired migration and invasion of HCC [38]. In accordance to these reports, the present study showed that BRD4 inhibition by JQ1 significantly inhibited SACC cell migration and invasion. Increasing evidence indicates that epithelial–mesenchymal transition (EMT) is an important mechanism for tumor metastasis [39–41]. Cancer cells undergoing EMT is characterized by loss of cell polarity, gain of spindle-shaped morphology and enhanced cell invasion [42]. This process involves down-regulation of epithelial genes such as E-cadherin [43], together with up-regulation of mesenchymal genes such as Vimentin [44]. Various transcription factors, such as Twist, can activate the EMT process [45]. BRD4 inhibition limited distal metastasis of colorectal cancer by regulating several key proteins including E-cadherin and Vimentin in the progression of EMT [29]. BRD4 inhibition by JQ1 has been demonstrated to control EMT and reduce migration and invasion abilities of human non-small cell lung cancer cells [46]. Considering the importance of EMT in tumor metastasis, we evaluated the expression of EMT genes in SACC cells to further identify the underlying mechanisms of the inhibitory effect of JQ1 on SACC cell invasion. The result showed that the protein levels of Vimentin and Twist were significantly down-regulated in SACC cells treated with JQ1, while the protein levels of E-cadherin were significantly up-regulated. These data indicate that JQ1 inhibits SACC cell migration and invasion via repressing the progression of EMT.

As for mechanism of the suppression of BRD4 by JQ1, it is generally agreed that JQ1 competitively bind to acetyl-lysine recognition pockets, displace BRD4 from chromatin, and reduce the expression of oncogenes, leading to cancer cell growth inhibition and apoptosis [47]. Consistent with this statement, Fiskus et al. [13] showed that treatment with JQ1 reduced the BRD4 occupancy at the promoters of c-myc, BCL-2, and CDK6 and attenuated the mRNA and protein expression of these associated genes in acute myelogenous leukemia (AML) blast progenitor cells (BPC). Consistently, in the present study, we found that the expressions of targets for BRD4, including c-myc and BCL-2, were significantly down-regulated in SACC cells treated with JQ1. However, the effect of JQ1 on BRD4 expression has been seldom studied. Fiskus et al. [13] showed that JQ1 had no effect on BRD4 expression. In contrast, our present experiment found that JQ1 down-regulated expression levels of BRD4 mRNA and protein in SACC cells. Similar result was also found in our previous study on oral squamous cell carcinoma Cal27 cells [48]. The mechanisms of JQ1 induced-BRD4 down-regulation still remains unclear.

In summary, our data show that BRD4 is an important transcription factor in SACC and BRD4 inhibition by JQ1 inhibits SACC cell growth and invasion. Thus, BRD4 may be a new therapeutic target for SACC patients. Further work will be needed to investigated the effect of JQ1 on SACC growth and invasion in vivo. And the clinical translational research about the application of JQ1 on patients with SACC will be a greater challenge for us.

## Methods

### Cell culture

The human normal epithelial cells (provided by Prof. Xunwei Wu, School of Stomatology, Shandong University) were cultured in the epithelial conditional medium (Gibico, Grand Island, NY, USA). Two SACC cell lines, ACC-LM and ACC-83 (provided by Shandong Provincial Key Laboratory of Oral Tissue Regeneration), were grown in high-glucose Dulbecco’s modified Eagle’s medium (DMEM) (Hyclone, Logan, USA) supplemented with 10% (v/v) fetal bovine serum (FBS; Gibco, Grand Island, NY, USA), 100 U/ml penicillin (Invitrogen, Camarillo, CA, USA), and 100 μg/ml streptomycin (Invitrogen) with 5% CO_2_ at 37 °C. In this study, cells were cultured in the medium supplemented with JQ1 (Selleck Chemicals, Houston, TX) at the concentrations of 0.1, 0.5 or 1 μM. Cells maintained in medium supplemented with 0.1% dimethyl sulfoxide (DMSO) were used as control.

### Cell proliferation assay

Cell Counting Kit-8 (CCK-8; Dojindo, Kumamoto, Japan) was used to detect the effect of JQ1 on cell proliferation according to the manufacturer’s instructions. Briefly, the human normal epithelial cells were seeded in 96-well plates at a density of 5000 cells/well. ACC-LM and ACC-83 cells were seeded in 96-well plates at a density of 3000 cells/well. These cells were maintained with various concentrations of JQ1, respectively. After cultured for 1–4 days, 10 μl of CCK-8 solution was added to each well, and the plates were incubated for 3 h at 37 °C. The optical density (OD) levels were measured at 450 nm using the SPECTROstar Nano microplate reader (BMG Labtech Inc., Ortenberg, Germany).

### Cell cycle analysis

The Cell Cycle and Apoptosis Analysis Kit (Beyotime, Shanghai, China) was used to evaluate the influence of JQ1 on cell cycle of the human normal epithelial cells, ACC-LM and ACC-83 cells. The cells were seeded in 6-well plates at a density of 2 × 10^5^ cells/well treated by JQ1 at various concentrations, respectively. After cultured for 24 h, cell cycle was measured by a flow cytometer (FACSCalibur, BD Biosciences).

### Annexin V/PI assays for apoptosis

The human normal epithelial cells, ACC-LM and ACC-83 cells were seeded in 6-well plates at a density of 2 × 10^5^ cells/well. JQ1 at the concentration of 1 μM was added in the medium. After 48 h incubation, the apoptosis of cells was evaluated using an Annexin V-FTIC/propidium iodide (PI) apoptosis detection kit (eBioscience, Vienna, Austria) according to the manufacturer’s instructions.

### Colony formation assay

ACC-LM and ACC-83 cells were seeded in 6-well plates at a density of 1000 cells/well and maintained in the medium with JQ1 at various concentrations. After cultured for 7 days, the cells were stained with crystal violet, photographed and counted.

### Immunofluorescence staining

Cells were cultured and analyzed by an Olympus immunofluoresecence microscope (Olympus, USA). In brief, ACC-LM and ACC-83 cells were cultured in the medium with JQ1 at the concentration of 1 μM. After 24 h, cells were fixed in culture well with 4% paraformaldehyde for 30 min. Then cells were permeabilized with 0.1% Triton X-100 for 10 min, blocked with 10% donkey serum for 1 h. And then, cells were stained with 1:200 primary rabbit anti-human BRD4 monoclonal antibody (Abcam, MA, USA) at 4 °C overnight. After washing three times with 1× phosphate buffered saline (PBS), cells were incubated with 1:200 goat anti-rabbit secondary antibodies (ZSGB-BIO ORIGENE, Beijing, China). 4, 6-diamidino-2-phenylindole (DAPI) at the concentration of 1 mg/ml was used for nuclear visualisation and it was added at the end of the process. Images were collected by fluorescence microscopy.

### Wound-healing assay

ACC-LM and ACC-83 cells were seeded in 6-well plates at a density of 2 × 10^5^ cells/well. After 24 h incubation, a denuded area was created across the diameter of dish by a yellow tip. And then the cells were washed with 1× PBS five times and incubated in a serum free high-glucose DMEM with JQ1 at various concentrations, respectively. Phase-contrast images were taken at a time point of 0 and 20 h of incubation. Images were analyzed with Image Pro Plus 6.0 software. The areas covered by migrated cells (wound recovery) was calculated.

### Transwell assay

Transwell assay was performed to evaluate the effects of JQ1 on the in vitro invasion of ACC-LM and ACC-83 cells using a 24-well Transwell plate (8 µm, Costar, Cambridge, MA, USA). A Corning^®^ Matrigel^®^ Basement Membrane Matrix (Becton–Dickinson & Co. Mountain View, CA) was plated on the upper surface of the Transwell plate to mimic the extracellular matrices underlying the cells in vivo. For each group, 5 × 10^4^ cells/insert were seeded on the matrix and incubated in 200 μl of serum free high-glucose DMEM containing JQ1 at various concentrations. The insert was placed on 24-well plate containing 600 μl of high-glucose DMEM complemented with 10% FBS as a chemoattractant. After 24 h incubation, the cells on the upper surface of the insert were removed gently with cotton-tipped swabs and the cells on the lower surface were fixed with 4% paraformaldehyde for 30 min at room temperature and stained with 0.1% crystal violet for 5 min. The number of cells was counted in five randomly selected fields, and the mean number of cells was calculated.

### RNA isolation and quantitative real-time polymerase chain reaction (qRT-PCR) analysis

ACC-LM and ACC-83 cells were seeded in 6-well plates at a density of 2 × 10^5^ cells/well and then maintained in medium supplement with JQ1 at various concentrations. Total RNA was extracted using Trizol^®^ reagent (TaKaRa Bio-tech, Tokyo, Japan) from cells. A Reverse Transcriptase kit (TaKaRa Bio-tech) was used for complementary DNA (cDNA) synthesis. qRT-PCR was performed using SYBR^®^ Primix Ex TaqTM kit (TaKaRa Bio–tech) according to the manufacturer’s instructions to analyze mRNA expression levels of BRD4. Relative fold levels were determined using the 2^−ΔΔCT^ method, with glyceraldehyde-3-phosphate dehydrogenase (GAPDH) used as housekeeping control. The sequences of the primers for amplification of human BRD4 and GAPDH were as follows: BRD4: 5′-ACCTCCAACCCTAACAAGCC-3′ and 5′-TTTCCATAGTGTCTTGAGCACC-3′; GAPDH: 5′-GCACCGTCAAGGCTGAGAAC-3′ and 5′-TGGTGAAGACGCCAGTGGA-3′.

### Western blot analysis

Cells were solubilized in radio-immunoprecipitation assay (RIPA, Beyotime) containing 1% phenylmethanesulfonyl fluoride (PMSF, Beyotime) for 30 min on ice followed by centrifuging for 10 min at 4 °C, 12,000*g*. The supernatant was run on 12% sodium salt-polyacrylamide gel electrophoresis (SDS-PAGE, Beyotime) and electrotransferred to 0.45 μm polyvinylidene fluoride (PVDF) membranes for 1 h at 100 V. The PVDF membrane was probed with primary antibodies overnight at 4 °C. Antibodies used for western blot analysis were as follows: (1) rabbit anti-human Cyclin D1 (1:1000, Abcam, Cambridge, MA, USA), (2) rabbit anti-human cl-C3 (1:1000, CST, Danvers, MA, USA), (3) rabbit anti-human Twist (1:1000, Abcam), (4) rabbit anti-human E-cadherin (1:1000, Abcam), (5) rabbit anti-human Vimentin (1:1000, Abcam), (6) rabbit anti-human BRD4 (1:1000, Abcam), (7) mouse anti-human c-myc (1:1000, Abcam), (8) mouse anti-human BCL-2 (1:500, Santa Cruz, CA, USA). And then the membranes were incubated in 1:5000 HRP-labeled goat anti-rabbit IgG (CST) or horse anti-mouse IgG (CST). The proteins were visualized using the Chemiluminescent HRP Substrate (Millipore, Billerica, MA, USA).

### Statistical analysis

All results were expressed as mean ± SEM from at least three replicates. One-way ANOVA and Student’s unpaired *t* test were used to analyze significance using the spss 16.0 software. Values of *P* < 0.05 were considered statistically significant.

## Abbreviations

BRD4: bromodomain-containing protein 4
SACC: salivary adenoid cystic carcinoma
CCK-8: Cell Counting Kit-8
qRT-PCR: quantitative real-time polymerase chain reaction
mRNA: messenger RNA
EMT: epithelial–mesenchymal transition
BET: bromodomain and extraterminal domain
Pol II: polymerase II
P-TEFb: positive transcription elongation factor complex b
HCC: hepatocellular carcinoma
cl-C3: cleaved caspase-3
DMEM: high-glucose Dulbecco’s modified Eagle’s medium
FBS: fetal bovine serum
DMSO: dimethyl sulfoxide
PBS: phosphate buffered saline
DAPI: 4, 6-diamidino-2-phenylindole
OD: optical density
PI: propidium iodide
cDNA: complementary DNA
GAPDH: glyceraldehyde-3-phosphate dehydrogenase
RIPA: radio-immunoprecipitation assay
PMSF: phenylmethanesulfonyl fluoride
SDS-PAGE: sodium salt-polyacrylamide gel electrophoresis
PVDF: polyvinylidene fluoride
VEGF: vascular endothelial growth factor
